# The Effect of Single or Repeated Home Visits on the Hanging and Use of Insecticide-Treated Mosquito Nets following a Mass Distribution Campaign - A Cluster Randomized, Controlled Trial

**DOI:** 10.1371/journal.pone.0119078

**Published:** 2015-03-16

**Authors:** Albert Kilian, Connie Balayo, Mitra Feldman, Hannah Koenker, Kojo Lokko, Ruth A. Ashton, Jane Bruce, Matthew Lynch, Marc Boulay

**Affiliations:** 1 Malaria Consortium, London, United Kingdom; 2 Tropical Health LLP, Montagut, Spain; 3 Malaria Control Program, Ministry of Health, Kampala, Uganda; 4 Malaria Consortium Uganda, Kampala, Uganda; 5 Johns Hopkins Bloomberg School of Public Health Center for Communication Programs, Baltimore, MD, United States of America; U.S. Naval Medical Research Unit Six (NAMRU-6), PERU

## Abstract

**Background:**

Study objective was to evaluate the effectiveness of commonly used post-campaign hang-up visits on the hanging and use of campaign nets.

**Methods:**

A cluster-randomized trial was carried out in Uganda following an ITN distribution campaign. Five clusters (parishes, consisting of ∼11 villages each) were randomly selected for each of the three study arms with between 7,534 and 9,401 households per arm. Arm 1 received one hang-up visit, while Arm 2 received two visits by volunteers four and seven months after the campaign. Visits consisted of assistance hanging the net and education on net use. The control arm was only exposed to messages during the campaign itself. Three cross-sectional surveys with a two-stage cluster sampling design, representative of the study populations, were carried out to capture the two key outcome variables of net hanging and ITN use. Sample size was calculated to detect at least a 15 percentage-points change in net use, and was 1811 at endline. The analysis used an intention-to-treat approach.

**Findings:**

Both hanging and use of ITN increased during follow-up in a similar way in all three study arms. The proportion of the population using an ITN the previous night was 64.0% (95% CI 60.8, 67.2), for one additional visit, 68.2% (63.8, 72.2) for two visits and 64.0% (59.4, 68.5) for the control. The proportion of households with all campaign nets hanging increased from 55.7% to 72.5% at endline (p<0.0005 for trend), with no difference between study arms. Financial cost per household visited was estimated as USD 2.33 for the first visit and USD 2.24 for the second.

**Conclusions:**

Behavior change communication provided during the campaign or through other channels was sufficient to induce high levels of net hanging and use and additional “hang-up” activities were not cost-effective.

## Introduction

Distributions of long-lasting insecticidal nets (LLIN), a sub-category of insecticide-treated nets (ITN), have now been widely accepted as one of the key interventions for malaria prevention and mass distribution campaigns as the best approach to achieve a rapid scale-up. However, a consistent gap between net ownership as defined by households owning at least one ITN and use by specific target groups or the general population has been observed which—at least to a large part—has been interpreted as a lack of ability or willingness to hang and/or use the nets [1–9]. Application of recently expanded indicators for ITN ownership and use [10] suggests that most of this gap was due to insufficient ITN within the household rather than unwillingness to use [11]. However, in the late 2000’s the main hypothesis was that difficulties in hanging the net and lack of knowledge about use were the key barriers to use [9]. This resulted in calls for active support in net hanging and inter-personal communication through home visits after the campaign distribution [1,4,5,9,12] and WHO now recommends to include “hang-up” activities by community volunteers as part of LLIN distribution campaigns [13]. Detailed instructions on the implementation of such activities have been issued [14] and included in many LLIN mass distribution campaigns [15–20].

In recent years there has been a significant increase in the literature on determinants of net use and reasons for non-use which have been shown to be complex, reflecting the living conditions of poor rural populations in high-risk malaria transmission areas. They include environmental factors such as climate and mosquito density, community norms and values, intra-family dynamics regarding decision taking and gender or age priorities, and factors associated with the net itself such as age or physical condition [21–27]. However, published data to date does not suggest that difficulty in hanging is a significant factor in non-use even though it is frequently mentioned in focus group discussions [21]. Cohee and coworkers studied ITN use among HIV affected households in Rakai District, Uganda, and found that only 6% of the non-users said the net was “too difficult to mount” [12]. From two post-campaign surveys in Niger Thwing and colleagues report that <5% of unused nets were not used due to difficulties in hanging [15]. In Kenya Alaii et al. [23] found 4% of the reasons given for non-use of ITN by children to be associated with technical difficulties in hanging, and from Malawi Holtz and coworkers [28] report 5% of non-users stating such difficulties. Five additional studies included in a review on quantifiable reasons for net non-use by Pulford at al. [24] make no mention at all of technical problems in hanging. On the other hand there is evidence that behavior change communication (BCC) without “hang-up” can significantly increase net use either through mass media [8], intensive and repeated inter-personal communication [28,29], or material incentives [30].

Published literature regarding the effects of door-to-door visits following an LLIN mass distribution campaign on net hanging and use rates is as yet very limited. The previously mentioned post-campaign surveys in Niger [15] found net use in children under five only increased by 3 percentage-points after intervention by Red Cross volunteers (72% vs. 74%) while in Togo [16], households with a volunteer follow-up visit showed an 8 percentage-point increase in nets hanging (80.4% vs. 72.3%) but only a 3 percentage-point increase in ITN use by children (89.0% vs. 86.2%) which was not statistically significant. In Madagascar 59 districts received an integrated LLIN mass campaign with volunteer door-to-door visits post-campaign and results were compared to districts with no campaign where ITN were obtained from routine distributions or from the retail market. ITN use among children in households with any ITN was 94.6% and 90.0% respectively, with the difference not statistically significant [31]. A post campaign survey in Luangwa District in Zambia [19] revealed no difference in the proportion of children using an ITN the previous night comparing ITN owning households where a community health worker had hung a net or not (53.5% vs. 54.5%). In the same district of Luangwa, Keating and coworkers undertook a quasi-experimental study of the effect of door-to-door interpersonal communication on ITN use [32] and found a strong increase in use in both intervention and control groups with no statistical difference at the end of the trial (82.8% vs. 79.8%).

Since the organization of door-to-door visits has its costs even when undertaken by community volunteers and hang-up activities are routinely undertaken by programs even though available evidence to support it is not very strong, more evidence is needed to judge whether this approach should continue to be recommended for use in all campaigns. This study was undertaken in Uganda within the context of the Alliance for Malaria Prevention Operational Research Working Group in order to produce more evidence regarding this question. A similar study was undertaken in Togo and will be reported on separately.

## Methods

### Study site

The study was carried out in Kamuli District within its January 2010 administrative boundaries. This location was selected in close collaboration with the National Malaria Control Program based on the following criteria: 1) a mass distribution of LLIN planned within the time window of the study; 2) an anticipated moderate net use rate based on the results of the 2009 Malaria Indicator Survey in the Eastern Region of Uganda [33] which showed 57.5% of children under 5 in households with at least one ITN using a net the previous night; and 3) no previous mass campaigns of LLIN.

**Fig 1:**
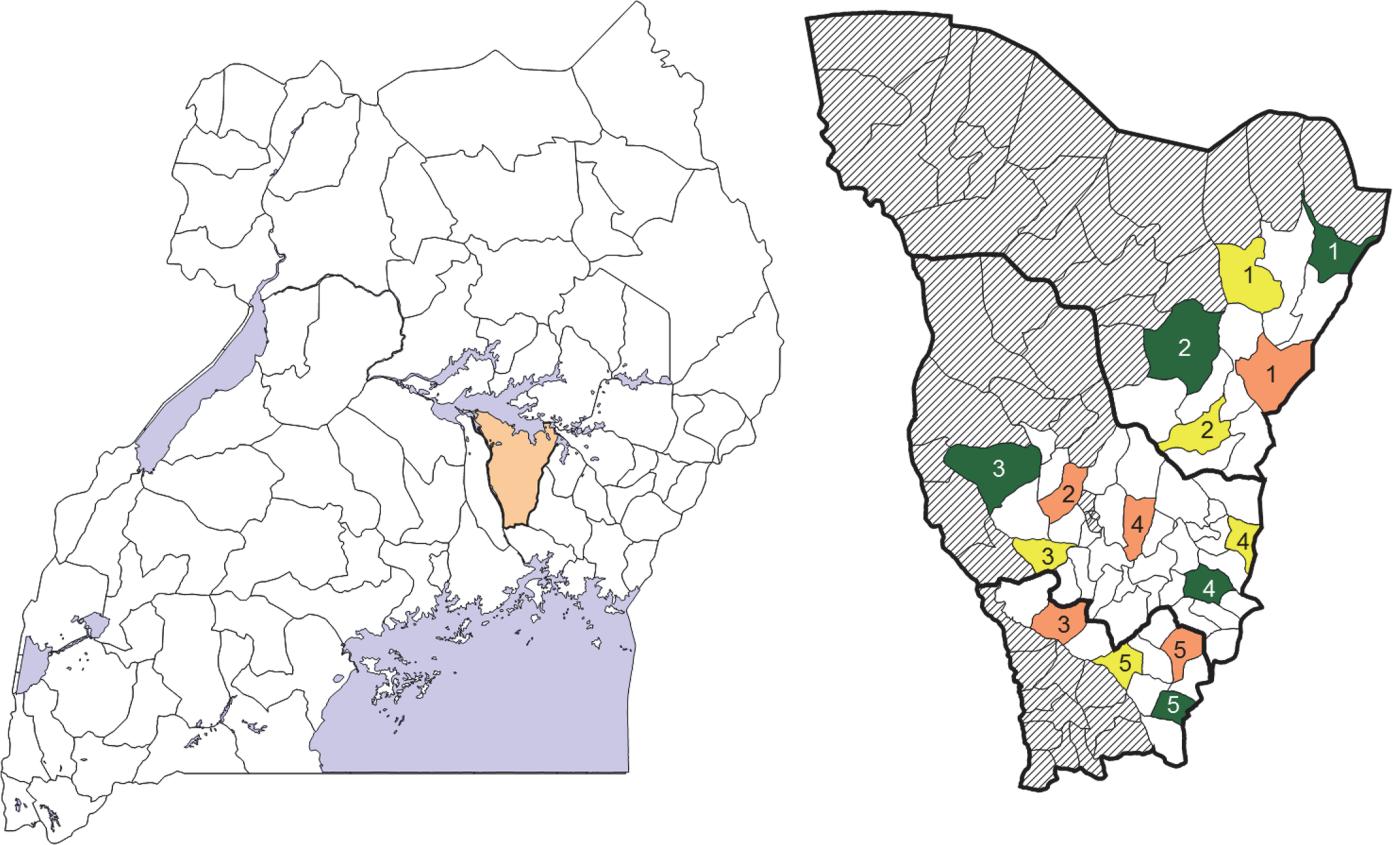
Map of Uganda with Kamuli District (2010 borders) and study clusters (parishes). Numbers represent the five geographical strata; orange: study arm 1; yellow: study arm 2; green study arm 3. Shaded areas were excluded from selection (details see methods section)

Kamuli District is bordered by Lake Kyoga to the North, the Nile River to the West, Jinja District to the South and Iganga and Kaliro Districts to the East (Fig. 1). Kamuli is part of the Busoga kingdom with a majority population of Basoga and minorities of Isiga, Banyoro and Bagungu ethnic groups. In addition to English Lusoga and Luganda are the main spoken languages. Kamuli is a predominantly agricultural district with rice, sweet potatoes and plantains being the main crops. In addition, fishing and animal husbandry are also significant sources of income. The estimated population of the district in its 2010 boundaries was 558,000 based on the 2002 National Census and approximately 707,600 in 2010 based on a 3% annual growth rate assumption.

### Hypothesis and study design

The hypothesis to be tested was that i) a single volunteer visit with the content as recommended by WHO (physical assistance with hanging, and educational messages on net use) would increase net hanging and use by at least 15 percentage points and ii) there would be a dose-response relationship with the number of visits by the volunteer, i.e. two visits would have a better effect than a single visit. The two major outcomes were defined as the proportion of households hanging all their nets obtained from the campaign and the proportion of the *de facto* population using an ITN the previous night if they had access to one.

**Fig 2:**
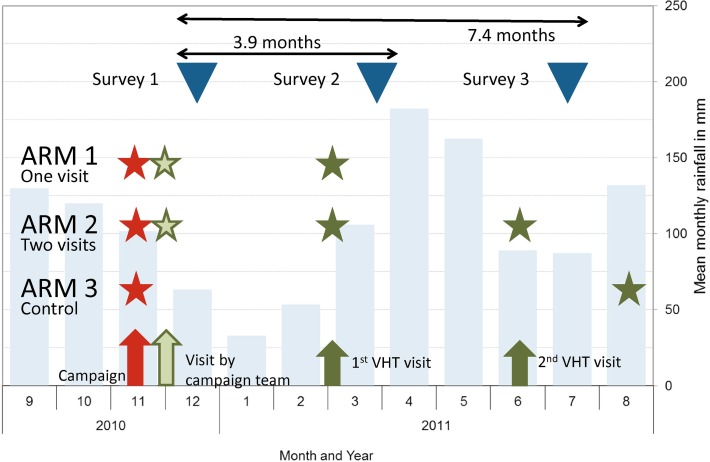
Rainfall pattern for Kamuli District and timing of study activities. Light blue bars represent monthly rainfall; red = LLIN mass campaign; green = interventions; stars show which timing of activities per study arm.

The design was a cluster-randomized, controlled study where the administrative unit of a parish (Local Council 2) was defined as cluster for randomization of interventions and control. In Kamuli District, a parish comprises on average 11 villages (Local Council 1) and 10,000 people and was the lowest unit at which training for the LLIN mass campaign was carried out. This approach excluded the use of villages as randomization clusters as it would have been impossible to avoid contamination of nearby control villages. First, sub-counties where a targeted LLIN distribution to young children had previously been carried out were excluded. Second, the district capital, Kamuli Municipality, was excluded so that all clusters had a rural background. Third, any parish bordering the Nile River or Lake Kyoga was excluded as these areas were anticipated to have higher levels of nuisance biting mosquitoes and hence a higher propensity to use nets. This gave a list of 47 Parishes eligible for selection. In the next step fifteen parishes (five clusters per study arm) were selected in such a way that no parish would directly border another eligible parish in order to minimize contamination between study arms. These parishes were then grouped into five geographical groups of three (Fig. 1) to ensure matching contribution of each sector of the district to each study arm. Within each of these groups parishes were randomly allocated to one of the study arms. A detailed list of selected parishes by study arm and group is provided in additional S1 File. The study participants were defined as all households residing in the 15 selected parishes with a total of 24,471 households: 7,534 in study arm 1, 7,535 in arm 2 and 9,401 in arm 3. The study arms were defined as follows (see also Fig. 2):

ARM 1: One “hang-up visit”; All households receive one visit by members of the Village Health Team (VHT) four months after the LLIN distribution. Visits consisted of physical assistance with hanging the net, and educational messages on net use.

ARM 2: Two “hang-up visits”; As arm 1 but with an additional, second home visit by the VHT seven months post distribution.

ARM 3: Control; No visits by the VHT for net promotion and hanging. However, after the final survey, VHT members conducted a hang-up visit.

As part of the standard operating procedures of LLIN campaigns in Uganda at the time the campaign teams were to visit some households in the two days following distribution to check on hanging of nets and give information. These visits were not part of the study interventions but were allowed in study arms 1 and 2. They were not carried out in the parishes of study arm 3.

### Mass distribution campaign

The mass campaign, funded by the Global Fund Round 7, targeted pregnant women and children under five with LLIN in all districts in the country. The Kamuli district LLIN distribution was conducted by the Ministry of Health (MoH) and a consortium of NGOs led by Program for Accessible Health and Education (PACE) with the final distribution occurring on December 1^st^ 2010. Households and their eligible population were registered in a house-to-house exercise prior to net distribution. All pregnant women were allocated one LLIN each and children under five were allocated LLIN in the ratio of one for every two children in the household. However, if there were three children under five, two nets were given. This allocation rule was used to determine the number of nets each household was to receive and this number was written on a coupon issued to the household to be redeemed at the distribution point. The date set for distribution was communicated to the heads of households instructing them where to collect their nets. In addition, radio announcements communicated the date and the distribution point. The registration exercise lasted two days, the actual net distribution one day. Before and during the collection process education was provided to the beneficiaries on malaria prevention and how to correctly hang a net and this was done in all three study arms.

### Home visits

The intervention home visits were done by VHTs and organized by the NGO PACE. A series of activities were carried out by PACE to prepare and ensure quality education and hang up activities in the households in arm 1 and arm 2. PACE developed a training curriculum based on the Red Cross guidelines for the hang-up intervention generally recommended by WHO. VHTs were selected and organized into teams and trained to follow a standard operating guideline for the hang-up and follow-up. The guidelines prescribed using a job aid to provide education on LLIN, asking about number and verify status of campaign LLIN in the household, noting how many LLIN are hanging, providing assistance with hanging LLIN and filling out monitoring forms for each household location and identifiers. Independent supervisors conducted quality checks of the intervention and verified the validity of data entry by the VHTs. The job aid used by the VHT is presented as additional S2 File.

Based on the PACE project records of the home visits undertaken, volunteers visited 87.9% of all eligible households for the first visit in study arm 1 and 78.4% for study arm 2. The second visit (arm 2 only) had a completeness of 83.9%.

### Evaluation surveys and sample size

For the evaluation of the interventions three cross-sectional household surveys were carried out using a two-stage cluster sampling design. The first survey served as baseline immediately after the mass distribution. The second and third surveys each followed two to four weeks after the home visit interventions in arms one and two (Fig. 2). Each study arm was considered a sampling domain and a sample of 30 villages was taken with probability proportionate to size (PPS) using the number of households recorded during the mass campaign as a measure of size. Within each village, 25 households were sampled with equal probability from the lists of all households registered during the mass campaign. Random numbers were generated by Stata 11 (StataCorp LP, College Station, Texas, USA) statistical software. Villages were maintained for each survey while households within villages were re-selected for each survey round from the same lists.

The sample size of 750 households per survey and study arm was calculated using the sample size module of the Stata software and applying an alpha error of 0.017 or 98.3% confidence interval (adjusting for multiple comparisons between study arms by dividing an alpha error of 0.05 by three), a beta error of 0.1 (power 90%), a design effect of 2.0, a 5% non-response rate, a net use rate at baseline of 50% and a difference between study arms of at least 15 percentage points. The total targeted sample was 2,250 households per survey round.

Pre-coded, structured questionnaires were used for data collection within households with the head of household or his or her spouse being the main respondent. In the first survey, the person going to the distribution point to pick up the net from the mass campaign was also interviewed. The questionnaire included a roster of all household members, a section on characteristics of the household and assets owned, a section on knowledge and perceptions around ITN, on the process of obtaining LLIN from the campaign including any home visits thereafter and hanging of the nets, and a roster of all nets found in the household. Each net was physically observed by the interviewer provided permission was given by the respondent. Visual aids with images of all available brands of LLIN (labels and packaging) were used to identify campaign nets (Netprotect, Bestnet\AS, Denmark) and other ITN in the houses. Verbal consent was obtained from each household respondent after adequate information on the rights to refuse or withdraw had been provided.

The survey team consisted of experienced field workers and interviewers who were able to communicate in the local languages Lusoga and Luganda. A three-day training preceded each survey round and during data collections supervisors checked all filled questionnaires for completeness and consistency. In addition, spot-checks were performed on 10% of interviews conducted by each field worker to validate the data collected.

### Costing data

To capture the cost of the interventions, a standardized spreadsheet was provided to the implementing agent (PACE) to capture all direct costs involved in each of the two rounds of home visits as well as indirect costs such as staff time and other contributions. The cost data collection followed the approach previously developed by WHO and Malaria Consortium as described by Kolaczinski et al. [34] and excluded any cost of the research itself.

### Data entry and analysis

A relational data base constructed in EpiData 3.1 was used for data entry with consistency checks incorporated. All records were double entered and any discrepancies validated from the original paper records.

Data were then transferred to Stata for data processing and analysis. Having used PPS to sample, the data can be considered self-weighted at the survey village level. However, to adjust for possible distortion of results by the difference of village sizes, standardized sampling weights were calculated. Analysis was done by an intention to treat approach based on study arm and independent of whether or not a home visit could be verified. Statistics were adjusted for the cluster sampling design (design effect) of the evaluation surveys by using Stata’s survey commands. A p-value of 0.017 was used as cut-off for statistical significance based on multi-comparisons between study arms (0.05 divided by 3).

The wealth index was computed at the household level using principal component analysis (PCA) [35]. The variables for household amenities, assets, livestock, and other characteristics that are related to a household’s socioeconomic status were used for the computation. All variables were dichotomized except those of animal ownership where the total number owned was used. The first component of the PCA was used as the wealth index. Households were then classified according to their index value into quintiles. Quintiles were calculated separately for each study arm. For analysis of individual members of the household or nets the quintile allocation of the household was applied. Concentration index was used to analyze outcome differences by wealth. Standard errors and confidence intervals for the concentration indices were calculated using the formula suggested by Kakwani et al [36].

The indicator of “access to ITN within the household” for individual household members was calculated as recommended by MERG [10]. First, an intermediate variable of “potential ITN users” was created by multiplying the number of ITN in each household by a factor of 2.0. In order to adjust for households with more than one net for every two people, the potential ITN users were set equal to the de-facto population in that household if the potential users exceeded the number of people in the household. Second, the access indicator was calculated by dividing the potential ITN users by the number of de-facto members for each household and determining the overall sample mean of that fraction.

Responses related to questions on IEC/BCC were recorded using a Likert scale, i.e. by asking respondents to choose on a scale of agreement. Response options were recoded to read 2 for “definitely could,” 1 for “probably could,” –1 for “probably could not”, –2 for “definitely could not”. Similarly, the responses were recoded to read 2 for “strongly agree,” 1 for “somewhat agree,” –1 for “somewhat disagree,” and –2 for “strongly disagree.”

To test for effects between study arms and time points (survey) for the major outcome of ITN use a ‘difference of differences’ analysis was used. This was done using generalized estimating equation (GEE) models with main effects of study arm and survey together with study arm-survey interactions. The models assume a binomial distribution with identity link and an independent correlation structure. The study arm-survey interaction terms describe the difference of differences effect. Each model accounted for survey villages as well as the randomization clusters (parishes) and the geographically matched groups of parishes by introducing them as random effects in the binomial model. Wealth quintiles were also included in the model as a potential confounder.

Data sets as well as full study protocol are available from the corresponding author on request.

### Ethical clearance

Ethical clearance was obtained from the Uganda Council for Science and Technology (ref. number SS 2397) and from the Institutional Review Board of the Johns Hopkins Bloomberg School of Public Health (IRB number: 3051). Both institutions waived the need for written informed consent allowing verbal consent instead.

## Results

### The sample

The number of completed interviews per survey round and reasons for non-response are presented in Table 1. Details of households included by randomization clusters are given in additional S3 File. The proportion of the targeted households reached (2250 per survey) reduced over the course of the study from 88.6% in the first survey to 84.6% and 80.5% respectively. This rather low completion rate was, however, not caused by refusals to participate but rather by a high proportion of households from the original campaign registration lists that could not be identified. This proportion was 10.2% initially and increased to 14.8% and 18.9% in the following surveys with no statistically significant differences between study arms. While the initial failure to identify households was mainly due to incorrect registrations (nonexistent names of household heads or double registration of same household under different names), the increase over the course of the study was caused by movement of households outside the study area.

**Table 1:** Survey coverage based on a target sample of 2250 households per survey (750 per arm).

	Survey 1	Survey 2	Survey 3
	December 2010	March 2011	July 2011
	N (%)	N (%)	N (%)
**Interviewed**			
Arm 1: one visit	662 (88.3%)	628 (83.7%)	623 (83.1%)
Arm 2: two visits	668 (89.1%)	628 (83.7%)	591 (78.8%)
Arm 3: control	656 (87.5%)	647 (86.3%)	597 (79.6%)
**TOTAL**	**1986 (88.2%)**	**1903 (84.5%)**	**1811 (80.5%)**
**Refused/incomplete**			
Arm 1: one visit	15 (2.0%)	1 (0.1%)	3 (0.3%)
Arm 2: two visits	4 (0.5%)	9 (1.2%)	8 (1.1%)
Arm 3: control	16 (2.1%)	5 (0.7%)	3 (0.4%)
**TOTAL**	**35 (1.6%)**	**15 (0.7%)**	**13 (0.6%)**
**Moved/Non-existent**			
Arm 1: one visit	73 (9.7%)	121 (16.1%)	125 (16.7%)
Arm 2: two visits	78 (10.4%)	113 (15.1%)	151 (20.1%)
Arm 3: control	78 (10.4%)	98 (13.1%)	150 (20.0%)
**TOTAL**	**229 (10.2%)**	**332 (14.8%)**	**426 (18.9%)**

Key indicators of household characteristics, ownership of ITN and campaign LLIN retention are summarized in Table 2. All indicators showed a slight decrease over time, which, however, was not statistically significant for any of the indicators. There were also no statistically significant differences found between study arms with the exception of household ownership of one ITN per two people which was approximately 10 percentage-points lower in arm 1 compared to the other two arms throughout all three surveys (p<0.001).

**Table 2:** Household characteristics and ITN ownership.

Characteristics by Survey	Survey 1	Survey 2	Survey 3
	Estimate (95% CI)	Estimate (95% CI)	Estimate (95% CI)
Mean Household size	6.4 (6.2, 6.6)	6.3 (6.1, 6.4)	6.1 (5.9, 6.3)
HH with child under 5	80.6% (78.4, 82.8)	77.6% (75.1, 79.9)	74.6 (72.1, 78.9)
HH owns any ITN	96.5% (95.3, 97.3)	95.8% (94.8, 96.2)	95.7% (94.7, 96.6)
HH owns 1 ITN / 2 people	48.5% (45.4, 51.5)	47.1%(44.1, 50.2)	46.1% (43.0, 49.2)
Retention of campaign nets	97.9% (97.4, 98.3)	95.2% (94.9, 95.8)	94.4% (93.7, 95.2)
**Characteristics at baseline by study arm**	**Arm 1**	**Arm 2**	**Arm 3**
Mean Household size	6.6 (6.4, 6.9)	6.4 (6.1, 6.7)	6.2 (6.0, 6.5)
HH with child under 5	85.3% (83.5, 87.5)	78.8% (75.3, 82.0)	78.0% (74.4, 81.2)
HH owns any ITN	95.7% (93.8, 97.0)	96.9% (95.2, 98.0)	96.7% (93.9, 98.3)
HH owns 1 ITN / 2 people	41.4% (37.0, 45.9)	54.5% (48.4, 60.5)	49.4% (44.4, 54.6)
Retention of campaign nets	97.4% (95.8, 98.5)	93.6% (89.6, 96.1)	95.8% (90.9, 98.1)

HH = household.

### Primary outcome 1: Hanging of campaign nets

#### Households with all campaign nets hanging

The “proportion of households with all nets obtained from the campaign hanging at the day of the survey” was chosen as the main outcome indicator. Results by study arm and survey are presented in Table 3. Overall the proportion significantly increased by 16.8 percentage points, from 55.7% (95% CI 52.2, 59.1) at the first survey to 67.6% (64.5, 70.7) at the second and 72.5% (69.2, 75.6) at the third (p<0.0005 for trend). Although hanging rates were consistently lower in the control arm (study arm 3) by 6–7 percentage-points for all three surveys, the rate of increase over time in the control arm was not different from the increases in the intervention arms (p = 0.4). In all clusters an increase from the first to the second survey was observed. Complete hanging of campaign nets was very equitable with a concentration index of 0.007 (-0.005, 0.018). The proportion of households that had none of their campaign nets hanging decreased from 14.6% (12.5, 17.0) to 10.9% (9.2, 12.9) and 7.3% (6.2, 8.6) in the three surveys respectively but again, the change over time was the same in all three study arms (p = 0.6).

**Table 3:** Proportion of households with any campaign nets that had all of their nets hanging on the day of the survey (OR = crude Odds Ratio).

	Survey 1	Survey 2	Survey 3
	December 2010	March 2011	July 2011
	% (95% CI)	% (95% CI)	% (95% CI)
**Arm 1: one visit**	58.0% (52.7, 63.1)	74.1% (68.7, 78.9)	77.2% (71.7, 81.6)
OR to first survey	n.a.	2.07 (1.49, 2.89)	2.44 (1.76, 3.40)
**Arm 2: two visits**	58.8% (51.3–65.8)	66.1% (60.6, 71.2)	74.0% (68.8, 79.3)
OR to first survey	n.a.	1.37 (1.00, 1.86)	2.00 (1.38, 2.90)
**Arm 3: control**	51.2% (45.5, 56.8)	63.5% (57.3, 69.2)	67.2% (60.8, 73.1)
OR to first survey	n.a.	1.66 (1.26, 2.18)	1.96 (1.35, 2.83)

#### Time when nets were hung

Immediately following the distribution, 20.7% (17.9, 23.9) stated they had hung their nets the same or next day, 79.7% (74.4, 84.2) within the first week and 82.5% (77.9, 86.3) within the first month with no difference between study arms (p>0.2).

#### Use of provided materials for hanging

Hanging was facilitated by the use of the package of nails/hooks and strings that was given out together with the LLIN at the campaign distribution points. In the first survey, 90.4% (95% CI 87.7, 92.6), of respondents attending the distribution site confirmed that they received the hanging tools with the nets. Of those receiving hanging materials, 79.9% also said they actually used them, with no differences in rates between study arms. Households that used the nails and strings were more than three times more likely to have any campaign net hanging (90.2% vs. 71.7%, crude Odds Ratio (OR) 3.6, (95% CI 2.4, 5.4)) and four times more likely to have all hanging (64.9% vs. 30.4%, OR 4.2, (3.0, 5.9). Many respondents, 42.8%, stated that they had seen a demonstration of net hanging and use and 12.6% mentioned having received an information leaflet. In both cases rates were the same in all three study arms but neither of these exposures showed an association with hanging of campaign nets.

#### Reported difficulties in hanging

Among all households with campaign nets, 12.3% said at the first survey they had difficulty hanging the nets. This rate declined to 7.1% and 5.3% in the subsequent surveys, respectively. The change over time was identical between the control and intervention arms of the study, as well as between the two intervention arms (p>0.2). Difficulties were primarily logistical in nature (no place or materials to hang). Interestingly, stating difficulties in hanging the nets did not prevent households from hanging their nets. Among those stating difficulties, 85.7% had any campaign nets hanging compared to 89.9% of those without difficulties in hanging (p = 0.02), and for hanging all campaign nets the respective figures were 55.8% and 67.1% (, p<0.0001). Both outcomes did not vary by survey or study arm. Also, there was no difference in the rate of reported difficulties in hanging between those households that had a VHT assist them in hanging (7.7%) and those that did not (8.3%).

#### Determinants of hanging

At the first survey 59.2% of the campaign nets were hanging over a sleeping place; 39.0% were hanging open and 20.2% were folded or tied up. A small percentage of nets (8.3%) were not hanging but available in the room, and 27.2% were stored. The remaining 5.3% were temporarily taken away or the location was unknown to the respondent. There was no statistically significant difference between study arms, but the hanging rate of campaign nets was significantly higher if the household did not have enough nets to cover all members (67.3%) compared to 57.9% if the household had just the right number of nets and 45.9% if the household had extra nets (p<0.0001).

Interestingly, the reason behind the observed increase in hanging of nets appears to come from nets taken out of storage and put to use. The proportion of campaign nets stored away was 27.2% initially and then decreased to 19.1% at survey 2 and 12.0% at survey 3. The storage rate declined in a similar fashion in all three study arms even though they tended to be highest in the control arm at each survey and the difference between any intervention and control was statistically significant for the second (15.2% vs. 25. 4%, p<0.0001) and third survey (9.9% vs. 15.4%, p = 0.001).

### Primary outcome 2: use of nets

#### Population using ITN

The “proportion of *de-facto* population using an ITN the night preceding the survey” was the key outcome indicator for use of nets, and results by study arm, survey and cluster were very similar to those seen for hanging of nets (Table 4). Overall ITN use increased by 8.9 percentage-points from 56.4% at the first survey to 61.7% in the second and 65.3% in the third survey (p<0.0005 for trend). At none of the surveys was there a statistically significant difference between the study arms (p>0.09) and the rate of increase was very similar between arms with the lowest gains seen in study arm 2 (two visits). ITN use was “pro-poor”, i.e. in all three surveys it was highest among the poorest wealth quintile and continuously decreased with increasing wealth resulting in an overall equity index of 1.21 and a concentration index of -0.038 (-0.042, -0.033).

**Table 4:** Proportion of the *de facto* population using an ITN the previous night (OR = crude Odds Ratio).

	Survey 1	Survey 2	Survey 3
	December 2010	March 2011	July 2011
	% (95% CI)	% (95% CI)	% (95% CI)
**Arm 1: one visit**	54.9% (50.5, 59.2)	60.8% (56.2, 65.2)	64.0% (60.8, 67.2)
OR to first survey	n.a.	1.28 (1.04, 1.57)	1.46 (1.23, 1.74)
**Arm 2: two visits**	61.4% (56.6, 66.0)	65.5% (61.5, 69.3)	68.2% (63.8, 72.2)
OR to first survey	n.a.	1.19 (1.02, 1.40)	1.35 (1.11, 1.64)
**Arm 3: control**	53.6% (48.7, 58.3)	59.4% (55.3, 63.4)	64.0% (59.4, 68.5)
OR to first survey	n.a.	1.27 (1.01, 1.60)	1.54 (1.24, 1.91)

#### Use of nets

The proportion of campaign nets used for sleeping the previous night increased from 62.9% in the first survey to 67.9% in the second and 73.3% in the third, again with no difference in the rate of increase between study arms. Overall, campaign nets were used more frequently than non-campaign nets, 89.0% vs. 71.3% (p<0.0001), but the strongest predictor of use in the univariate analysis was whether or not the net was hanging over the sleeping place: if the net was hanging openly it was used in 96.6% of cases, compared to 94.0% if the net was hung tied up or folded and 38.3% if it was in the room but taken down.

#### Reasons for non-use of nets

Respondents were probed on reasons for non-use of nets and valid answers were provided for 86.6% of the non-used nets. Responses were grouped into two main categories: subjective reasons that are prone to be influenced by behavior change communication such as fear of side effects, inability to hang the net or perception that there is no malaria, and objective reasons that are more difficult or impossible to address, such as the usual occupant or the net not being present or available that night, the net being too old and torn, or having an excess net for which there is currently no use. A detailed list of categories is shown in Table 5 which presents results from the final evaluation at survey 3, i.e. after all interventions had been implemented. In all three study arms “net not needed”, i.e. an excess net for which there currently is no user, was the most common reason for not using the net, at 42.1%. The second most common reason was that the net was “too old, torn or dirty”, at 16.7% followed by “user not around” with 13.0%, and “net not available” with 8.4%. Together these reasons, categorized as objective, comprised 80.2% of the stated reasons for non-use compared to 9.9% for subjective reasons, among which “not ready to use net at this time” was the most common with 4.1% followed by “fear of side effects/dislike of net” with 2.7% and “no malaria/mosquitoes” with 2.6%. Inability to hang the net was the least mentioned reason for non-use with 0.6%, and all of these came from the control arm. The remaining 9.5% of responses were “other” without specification and, therefore, could not be categorized further. There was no statistically significant difference in reasons for non-use between the study arms at survey 3 (p = 0.3). Non-use due to the “net not being needed” was also particularly high for nets from households that were oversupplied with ITN (56%), i.e. had more than one ITN for every two people. This suggests that the newer campaign nets were kept for later use if more than enough nets were available.

**Table 5:** Reasons for non-use of ITN: final evaluation at survey 3 (N = 1391).

	Arm 1	Arm 2	Arm 3
	One visit	Two visits	Control
	% (95% CI)	% (95% CI)	% (95% CI)
	n = 406	n = 473	n = 512
**Subjective reasons**			
No malaria, no mosquitoes	1.7% (0.6, 4.5)	3.1% (1.1, 8.6)	2.7% (0.9, 8.1)
Fear side effects, dislike of net	4.1% (2.3, 7.3)	1.6% (0.7, 3.4)	2.6% (1.2, 5.5)
Not yet ready/willing to use net	5.8% (2.4,13.3)	2.6% (0.7, 9.2)	4.0% (1.9, 8.6)
Can’t hang net	0	0	1.3% (0.5, 3.7)
**Total**	**11.6% (7.0, 18.7)**	**7.3% (3.8, 13.6)**	**10.7% (6.8, 16.4)**
**Objective reasons**			
Too old/torn, dirty, used otherwise	20.2% (15.6, 25.9)	16.5% (11.0, 24.0)	14.5% (10.7, 19.3)
Net was not available last night	12.1% (9.8, 16.3)	6.6% (4.1, 10.3)	7.3% (5.2, 10.1)
User did not sleep here last night	11.7% (7.6, 17.7)	15.9% (10.3, 23.6)	11.8% (8.6, 15.8)
Net not needed at the moment	33.4% (27.3, 40.2)	45.1% (36.5, 54.0)	45.6% (39.0, 52.3)
**Total**	**77.6% (71.5, 82.6)**	**84.1% (77.5, 89.0)**	**79.1% (73.2, 83.9)**
			
**Other reasons** (not specified)	10.8% (6.8, 16.9)	8.6% (5.7, 12.9)	10.2% (7.1, 14.4)

#### Population with access to ITN within household and use gap

**Fig 3:**
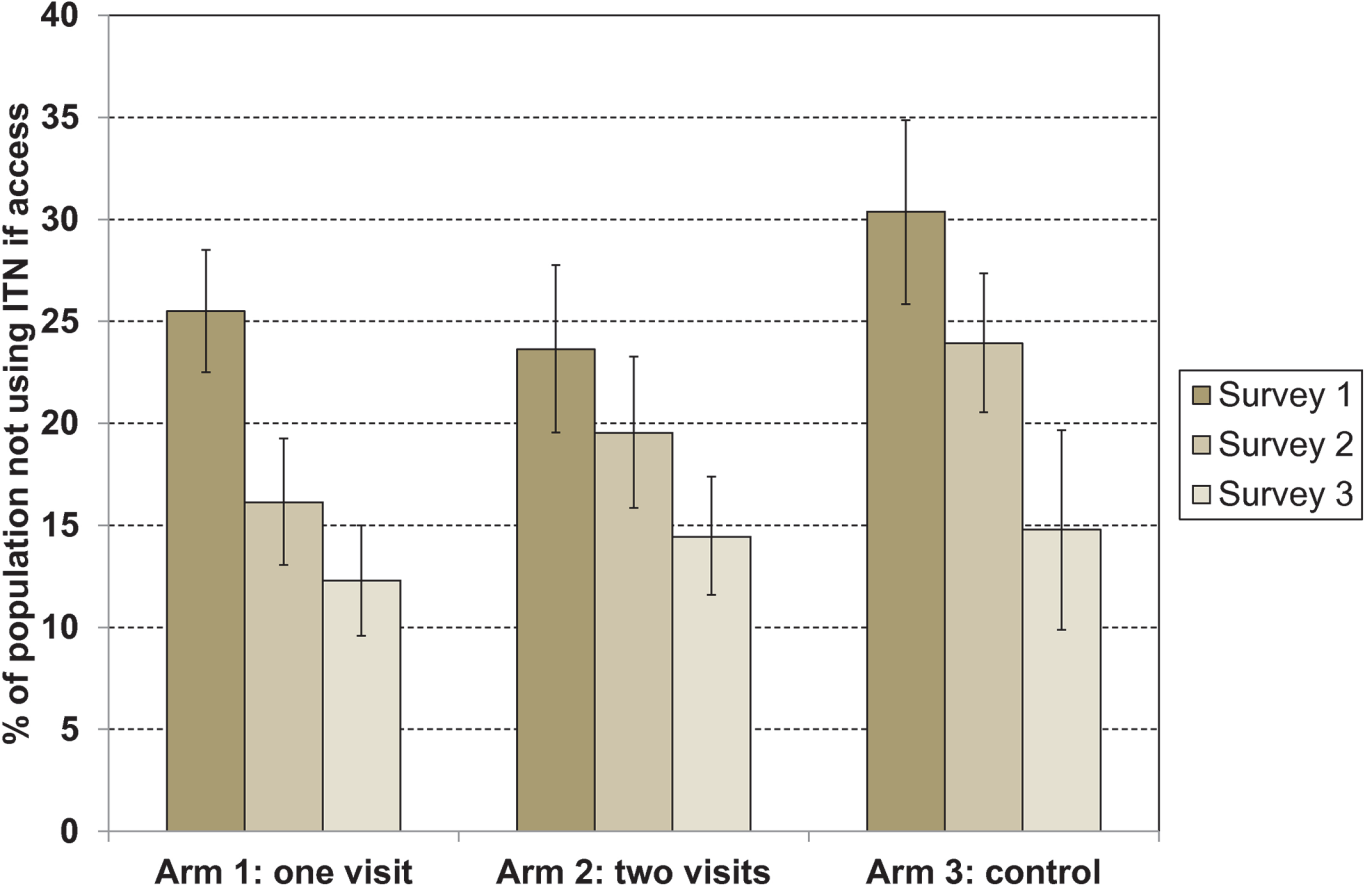
Use gap defined as population with access to an ITN but not using an ITN by survey and study arm. Error bars represent 95% confidence intervals.

While looking at the proportion of nets used is a good approach to explore the link between hanging, use, and reasons for non-use, the most critical criteria for population use of ITN is access to an ITN within their household. Overall 76.7% of the population had access to an ITN with no variation between surveys, but a consistently higher rate was reported in study arm 2, with 80.5%, compared to 73.1% in study arm 1 and 76.8% in study arm 3 (p = 0.001). Comparing use to access then defines the use gap, i.e. the proportion of the *de-facto* population with access to an ITN within the household not using it the previous night. Results are presented in Fig. 3 and show a similar pattern as seen in previous results: a continuous decrease of the use gap over time with a similar magnitude of change in all three study arms. Overall the use gap significantly declined from 26.5% in survey 1 to 20.2% in survey 2 and 13.8% in survey 3 (p<0.01), implying that at the final evaluation, 86.2% of those with access to an ITN also used it.

### Recall of hang-up visits by respondents

Table 6 summarizes the recall of the hang-up visits by the respondents and compares those results with the intervention coverage according to implementation records. Immediately following the campaign, the routine visit by the campaign team was only acknowledged by 14% in arm 1 and 16% in arm 2 while 3% in the control arm stated that they had received a visit regarding nets. For the study intervention, recall of the first hang-up visit was 46% and 45% in arms 1 and 2, respectively, and 9% for the control arm. For the second visit (arm 2 only), recall was 38% but still 22% in arm 1 (which had no second visit) and 13% in the control arm. Overall recall of a net-related hang-up visit among those who should have received the intervention (visit 1 or 2) was 42.8%, with the highest rate observed if the spouse of the head of household was the respondent (46.3%), followed by the head of household (40.7%) and other respondents (27.7%, p<0.0005). Recall also varied by education, with 32.9% of those without any schooling mentioning a hang-up visit compared to 46.4% for those with any school attendance.

**Table 6:** Recall of hang-up visit intervention.

	Routine post-campaign visit	Visit 1	Visit 2
	% (95% CI)	% (95% CI)	% (95% CI)
**Arm 1: one visit**			
Survey	13.9% (9.3, 20.3)	45.5% (39.8, 51.4)	21.8% (17.1, 27.4)
Implementation records	n.a.	87.9%	0%
**Arm 2: two visits**			
Survey	16.0% (10.7, 23.3)	44.5% (37.5, 51.7)	38.1% (32.8, 43.7)
Implementation records	n.a.	78.4%	83.9%
**Arm 3: control**			
Survey	3.3% (4.0, 10.9)	9.1% (7.1, 11.7)	12.8% (9.7, 16.6)
Implementation records	n.a.	0%	0%

Based on self-reporting by respondent (survey) and records from the intervention implementing agency.

Among the households in study arms 1 and 2 who confirmed that they had received the hang-up visit from the VHT (n = 806; 574 in survey 2, 232 in survey 3), 90.8% confirmed that the VHT had emphasized net use and that nets should be used every night, 83.8% said the VHT had mentioned hanging of nets and how to do it, 59.9% recalled the VHT talking about washing of and care for the net and 20.8% remembered a message about the importance of malaria prevention for health. Actual physical assistance in hanging the nets was reportedly provided to 18.3% of these households during the hang-up visit while 56.1% said the nets were already hanging. The remaining 25.6% only mentioned that the VHT did not assist without giving a reason. The majority of respondents who confirmed the intervention (91.6%) felt that the VHT had done a good job during the visit. These performance estimates did not vary between study arms, geographical randomization clusters or wealth quintile.

### Impact assessment for interventions

Statistical analysis based on the “difference of differences” analysis of the changes in ITN use between study arms and surveys adjusting for confounders and random effects in the GEE model is presented in Table 7. As already suggested by the very similar changes in the unadjusted ITN use gap (Fig. 3), none of the comparisons showed a statistically significant impact of the interventions (p>0.3).

**Table 7:** Difference of differences for ITN use by *de facto* population, adjusting for study design.

Comparison	Survey 2 against Survey 1	Survey 3 against Survey 2	Survey 3 against Survey 1
**Arm 1 vs. arm 3**			
**One visit against control**			
% change (95% CI)	0.7% (-5.2, 4.8)	-1.5% (-7.2, 4.5)	-0.9% (-7.0, 5.6)
p-value	0.851	0.615	0.777
**Arm 2 vs. arm 3**			
**Two visits against control**			
% change (95% CI)	-2.0% (-7.9, 4.3)	-1.9% (-7.5, 4.0)	-4.0% (-9.9, 2.3)
p-value	0.527	0.519	0.296
**Arm 1 vs. arm 2**			
**One visit against two visits**			
% change (95% CI)	2.7% (-3.3, 9.2)	0.3% (-4.4, 5.2)	2.9% (-2.9, 9.1)
p-value	0.380	0.904	0.325

### Behavior Change Communication and its effects

Since the intervention of VHT home visits did not appear to have caused the observed increases in net hanging and use, other aspects of Behavior Change Communication (BCC) were explored.

#### Exposure to messages and content recall

Respondents were asked whether they had heard or seen any messages on net hanging or use around the time of the mass campaign. At the survey immediately following the campaign 68.1% confirmed exposure to such messages with no difference between the study arms (p = 0.3) and no change was detected in the two follow-up surveys (p = 0.9). The most commonly mentioned sources were local leaders with 58.5% and health workers, 30.5%. Messages heard on radio played a lesser role, at 15.9%, from the campaign mobilization team, at 13.4%, and from family and friends, at 12.2%. The campaign leaflet was mentioned by only 4.0% while newspaper, drama shows and religious groups were mentioned by less than 1%. Two thirds (65.7%) of those exposed to messages mentioned a single source while 34.2% mentioned two or more sources with a maximum of four. The most commonly recalled message content was “hang your net” mentioned by 56.9% and “use your net” or “use net every night” with together 54.2%. About one third, 32.4%, mentioned “air your net before using”, and 24.7% remembered that “nets prevent malaria”. There was no difference between study arms neither for information sources nor for messages recalled.

#### Multivariate analysis of determinants for use

In order to explore how factors related to BCC impacted on ITN use, a multi-variable logistic regression analysis was undertaken with population use of ITN the previous night as the outcome variable (details are provided in additional S4 File). Controlling for survey, study arm and the geographical groups of clusters, the two most important determinants of individual ITN use were “intention to use nets every or most nights” expressed by the household respondent with an adjusted Odds-Ratio (OR) of 3.22 (95% CI 2.75, 3.78) and the household having at least one ITN for every two people, OR 2.76 (2.44, 3.12). The household respondent being confident to take action in preventing malaria (mean action score >1) was also positively associated with ITN use, OR 1.23 (1.10, 1.36), as was having discussed net use in the family, OR 1.25 (1.13, 1.38). High level of agreement with statements about the threat of malaria were negatively associated with ITN use, OR 0.81 (0.71, 0.92), implying that with increasing ITN use, the perceived threat decreased. Other factors significantly associated with ITN use were gender, with an adjusted OR for females compared to males of 1.16 (1.10, 1.22), wealth, with decreasing use as wealth increased (p = 0.0001), and age (p<0.0001), with highest use in under-fives and lowest use in the age groups 10–14 and 15–19 years. Education level of head of household or family size did not have an independent effect on ITN use in this model. However, overall, the final model only explained 12% of the variation of ITN use (R-squared 0.12) suggesting that other unmeasured factors also play a significant role.

### Cost of intervention per household reached

The financial cost of the intervention of one or two additional home visits to enhance ITN hanging and use after the campaign is shown in Table 8. For the first hang-up visit (study arms 1 and 2) the cost per household visited was USD 2.33 and for the second visit (study arm 2 only), USD 2.24. The relative share of indirect cost increased from 12% for the first visit to 27% for the second as these were mainly fixed costs and the number of visits was halved during the second visit. The highest single cost position were the expenses for the hang-up visits themselves (allowance for the Village Health Team members), which were USD 0.67 per household visited for the first and USD 0.82 for the second.

**Table 8:** Cost of hang-up intervention per home visited.

Cost category	1^st^ visit		2^nd^ visit	
	Cost/visit in USD	%	Cost/visit in USD	%
**Direct Cost**				
Sensitization	0.43	18.3%	0.09	4.0%
Training of Trainers	0.08	3.2%	0.12	5.5%
Training VHT	0.34	14.7%	0.40	18.0%
Hang-up visits	0.67	29.0%	0.82	36.7%
T-shirts	0.32	13.6%	0.00	0.0%
Data entry	0.02	1.1%	0.06	2.6%
***Sub-total***	***1*.*86***	***79*.*8%***	***1*.*49***	***66*.*8%***
Administrative fee 10%	0.19	8.0%	0.15	6.7%
**Total direct**	**2.05**	**87.8%**	**1.64**	**73.4%**
**Indirect cost**				
Operating	0.17	7.3%	0.35	15.8%
Staff	0.10	4.1%	0.20	8.9%
Audit	0.02	0.9%	0.04	1.9%
**Total indirect**	**0.28**	**12.2%**	**0.59**	**26.6%**
**Total cost**	**2.33**	**100.0%**	**2.24**	**100.0%**

## Discussion

Over time, the study found increasing rates of hanging and use of nets and a significant reduction of the use gap (those with access to an ITN within the household not using it) from 26.5% to 13.8%. But there was no difference detected in the outcome between the control and either intervention arm, nor between the two intervention arms themselves. This result is very similar to the only other study on net use and home visits with an experimental design from Luangwa, Zambia, although due to an error in the allocation of intervention and control clusters it was declared quasi-experimental [32]. In Luangwa District, home visits and interpersonal communication by community volunteers were provided in addition to the information given during the campaign over an 18 months period. This intervention resulted in a general increase of net use in children under five from 54% to 81%. But there was no difference detected between intervention and control (82.8% vs. 79.8%), and both intervention and control households had a high exposure to information on net use from the media (94.2% vs. 86.6%). Other published data comes from observational studies from Zambia [11], Niger [15], Togo [16] and Madagascar [31], and none of them found a significant impact of post-campaign home visits on ITN use, nor did any of the differences exceed 5 percentage-points. This suggests that improvements detected in our study were achieved by the campaign-related information and messages in combination with BCC through multiple media channels. This is plausible as achievements of the magnitude observed in this study have been shown in Cameroon to be possible by a well-organized media campaign alone with an increase of 12 percentage-points in net use by children and 7 percentage points for the general population [8]. An even larger increase attributable to BCC messages of 29% was reported from Zambia [37].

Data from this study strongly suggest that difficulties in hanging of nets is not the primary reason for non-use, and that nets are being hung and used if there is motivation and opportunity to use. Categorizing reasons for non-use in this study following suggestions by Alaii et al. [23] and Pulford et al. [24], the vast majority (74% at the first and 80% at the third survey) could be categorized as “objective”, i.e. not easily influenced by BCC, and only 18% and 10%, respectively, as subjective. Among subjective reasons, inability to hang the net was only stated for 3.5% of unused nets at the first survey and 0.6% at the final evaluation with no differences detected between study arms. Among the objective reasons, “net not needed”, i.e. a net without an evident user, was most common. Second, only 12% of households stated that they had difficulties in hanging the nets at the first survey reducing to 5.3% in the third survey and the reduction was similar in all study arms. Furthermore, stating difficulties in hanging did not prevent households from hanging nets as 86% still had at least one of the campaign nets hanging and 56% all campaign nets, compared to 90% and 67% respectively if no difficulties were stated. Third, even in the control group, gains in campaign nets hanging were observed with nets moving from being stored to being hung during the seven months of follow-up. This appears to be based on the exposure to messages during the campaign—mainly from local leaders (59%) and health workers (31%) and to a lesser degree from radio (16%) and family and friends (12%)—and assisted by the provision of a pack of nails and strings together with the campaign nets. The multi-variable regression analysis showed that the intention to use a net every or most nights was the strongest determinant of use (OR 3.2), followed by having enough nets for all household members (OR 2.8), discussing net use in the family (OR 1.3) and a high level of confidence to take actions for malaria prevention (OR 1.2). Intention to use nets every night or most nights and discussing net use with the family were both significantly associated with exposure to messages. At the same time the perceived threat of malaria decreased with increasing net use, suggesting that the experience of improvements in the malaria situation through net use encouraged continued use.

Taken together, this implies that cause and effect between hanging of nets and their use starts with exposure to messages which enhance the intra-household discussion about net use and willingness and confidence to use, leading to the ability to overcome any perceived or existing challenges in hanging of nets, and resulting in increased levels of net use if there are enough nets in the household. Repeated net use and the positive experience of a declining threat then further enhances net use with increasing use rates also among population groups that are usually less likely to use such as older children and adolescents [18, 20, 32]. This interpretation is in keeping with the Health Belief Model [38, 39], a widely used model in public health, which states that the combination of the perception of risk and beliefs about the barriers to and benefits of a health action determine health behaviors.

In the Ugandan setting, some level of net use and communication around nets has been ongoing for several years. It appears that the exposure to routine BCC around the campaign was sufficient to trigger this process and the additional intensive home visits by VHT members four and seven months after the distribution did not have an additional effect. At costs of USD 2.33 per household for the first visit and USD 2.24 for the second visit, this approach does not suggest good value for money considering that a mass media campaign to promote ITN use in Cameroon was costed at USD 0.16 per adult reached and USD 1.62 per additional person protected by a net [8].

Several limitations of the study need to be taken into account. First, although records from the NGO implementing the home visit intervention indicate that between 78% and 88% of households in the communities sampled for the evaluation surveys were visited by the VHT during the first and second round of intervention, this could not be verified by recall of the respondents. Only 46% and 45% of households in study arms 1 and 2, respectively, recalled the first home visit and 38% the second (study arm 2 only). One possible explanation could be that the respondent to the questionnaire might not have been present when the VHT member visited. However, this is not likely to explain most of the deficit in recall as a senior member of the household had to confirm the visit by fingerprint. Another reason could be confusion with activities of the VHT regarding health aspects other than malaria. The majority of households in this study that recalled a Hang-up visit regarding nets confirmed that the volunteer had talked to them about the importance of using the ITN (91%) and 84% about hanging the net, even though only in 18% did they actually assist in hanging. In connection with the high level of coverage from the implementer’s records this strongly suggests that the intervention was delivered in sufficient quality and quantity so that an effect could have been seen.

There was also a recall of a hang-up visit in the control arm by 9% and 13% of households at the second and third survey, respectively. This could either be due to confusion with other health activities by the VHT, or reflect home visits of volunteers undertaken on their own initiative, or be a recall error. Actual contamination by the organized intervention is highly unlikely based on the study design of using parishes as units of randomization and leaving at least one parish as a buffer zone between intervention and control clusters. Although a slight dilution effect on the assessment of the intervention cannot be excluded, the magnitude would not have been sufficient to change the key findings of this study.

The non-response rate in the evaluation surveys was higher than the usually tolerated 5% with 12% reported in the first survey, 16% in the second and 19% in the third. However, the rate of refusal by households was very low with a maximum of 1.6% in the first survey. Instead, most of the “non-response” was due to households on the list that were found to be nonexistent (false name) or misleading (household members registered as separate households). This comprised 10% of sampled households in the first survey. The further increase to 15% and 19% in the following surveys was mainly due to movement of households to other communities.

Measurement of the key outcome, the use of an ITN the previous night, was by recall by the respondent regarding who used which net, with a cross-check of these names against the list of household members. This is general practice for Malaria Indicator and Demographic and Health Surveys [40] but could possibly overestimate the true use rate if respondents exaggerate actual use based on perceived expectations. There is only one published study where ITN use was physically confirmed by a household visit in the early morning [23], and 72% of the population was found using an ITN, but this result was not compared against a self-reported use rate. In this study, the changes over time in reported ITN use were closely matched by observed changes in hanging nets, which makes it highly unlikely that the detected increases in use were caused by an increasing overestimation of ITN use rather than actual use.

Finally, this study was designed to measure the impact of one or two additional post-campaign home visits on net hanging and use based on standard procedures in the given setting of Uganda. The extent to which the findings reported here can be extrapolated to other settings where the level of pre-existing ‘net culture’ and intensity of BCC activities around a net campaign are different remains unclear. Results from a similar study in Togo published recently [41] show generally similar results although a significant increase in ITN use among children under five was found. This is a limitation with respect to the practical applicability of the study results for program managers, and can only be addressed once comparable evidence from more settings becomes available and can be subjected to a meta-analysis.

In conclusion, in the setting of Kamuli District, Uganda, behavior change communication provided during the LLIN mass campaign or through other channels was sufficient to induce high levels of net hanging and use and additional “hang-up” activities based on the recommended “home-visit” procedures did not provide any additional impact on net use, were not cost-effective and should not be recommended for similar settings.

## Supporting Information

S1 File List of clusters (Parishes) per study arm. (DOCX)

S2 File visual aid for the intervention home visits. (PDF)

S3 File Composition of final sample by study arm and intervention cluster. (DOCX)

S4 File Determinants of ITN use from multi-variable analysis. (DOCX)
