# Supplementary material for: The Effect of Single or Repeated Home Visits on the Hanging and Use of Insecticide-Treated Mosquito Nets following a Mass Distribution Campaign - A Cluster Randomized, Controlled Trial
Source: PLoS One. 2015 Mar 16;10(3):e0119078. doi: 10.1371/journal.pone.0119078 (PMC4361725; doi:10.1371/journal.pone.0119078)
Supplement: S1 File — (DOCX) [file pone.0119078.s001.docx]

**Additional file S1: List of clusters (Parishes) per study arm** (see Figure 1)

| **Parish** | **Subcounty** | **Group** | **Study arm** | **# of villages** | **Registered households in campaign** |
| --- | --- | --- | --- | --- | --- |
| Gumpi | Bugaya | 1 | 1 | 12 | 2094 |
| Kabukye | Kagulu | 1 | 2 | 11 | 1924 |
| Irundu | Kagulu | 1 | 3 | 12 | 1856 |
| Nakulyaku | Nabwigulu | 2 | 1 | 8 | 1510 |
| Gwase | Bugaya | 2 | 2 | 8 | 1550 |
| Bugaya | Bugaya | 2 | 3 | 13 | 2983 |
| Buluuya | Mbulamuti | 3 | 1 | 11 | 967 |
| Butansi | Butansi | 3 | 2 | 10 | 1007 |
| Kasozi | Namasagali | 3 | 3 | 11 | 1731 |
| Butende | Kitayunjwa | 4 | 1 | 14 | 1126 |
| Kidiki | Namwendwa | 4 | 2 | 4 | 768 |
| Makoka | Namwendwa | 4 | 3 | 8 | 1420 |
| Bugulumbya | Bugulumbya | 5 | 1 | 17 | 1837 |
| Kasambira | Bugulumbya | 5 | 2 | 13 | 2287 |
| Lulyambuzi | Wankole | 5 | 3 | 12 | 1411 |
