# Supplementary material for: The Effect of Single or Repeated Home Visits on the Hanging and Use of Insecticide-Treated Mosquito Nets following a Mass Distribution Campaign - A Cluster Randomized, Controlled Trial
Source: PLoS One. 2015 Mar 16;10(3):e0119078. doi: 10.1371/journal.pone.0119078 (PMC4361725; doi:10.1371/journal.pone.0119078)

HOW TO USE LLINs?

- LLINs should be used by everyone, every day, every night around the year. (If nets are not enough for everyone, children less than five years and pregnant women should get to use first)

IMPORTANT

- Before first using the net, hang outside for a full day to balance the insecticide. If not, it might cause eye or skin irritations
- Use far away from lighted candles or fires

The nets are rectangular to match with your sleeping space or bed. Use the four loops on the net to tie it up. Before sleeping every night, lower it to cover the mat, bed or cot and tuck the edges under it to keep mosquitoes away.

You can hang it in whatever works well for your sleeping place, such as:

1. Use sticks attached to the bed at each corner and hang from them
2. Hang it from nails sunk in the walls, on strings or poles that you can run across your room
3. Any other way that works best for your home

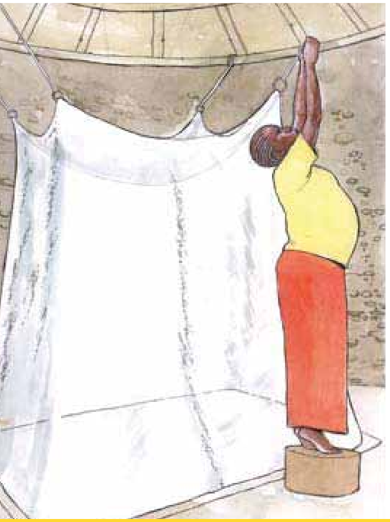

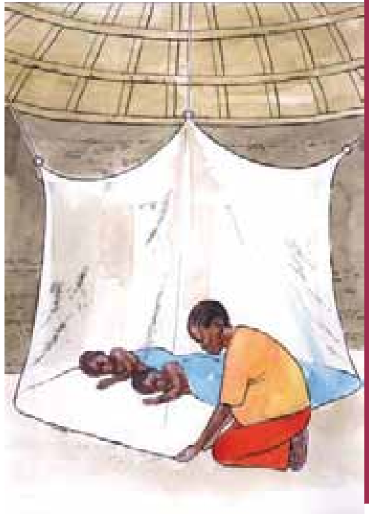

HOW TO CARE FOR LLINs?

- Wash when dirty, but only once in three months
- Don't use strong soap as this will wash away the insecticide and make it less powerful
- Check regularly and sew holes or repair damage immediately, if you see any
- Lift it up in day time and knot it above bed or cot, so it won't get ripped and torn
- Replace if torn or damaged beyond repair

8 Reasons why everyone, every day, every night should use LLINs

1. Totally safe for men, women including pregnant mothers, unborn babies, infants and children
2. Repels and kills mosquitoes, hence most effective in preventing malaria
3. Daily use cuts down malaria attacks by more than half, saving time and money treating malaria
4. High quality nets recommended by WHO - provided free of charge by Ministry of Health, Government of Uganda
5. Needs no retreatment as insecticide lasts for 3 to 5 years
6. Does not harm children on accidental chewing
7. Does not choke as small holes allow for air to flow in easily (daily use eases any initial discomfort)
8. Does not cause sickness or infertility

VHT job aid for LLIN distribution follow up visit

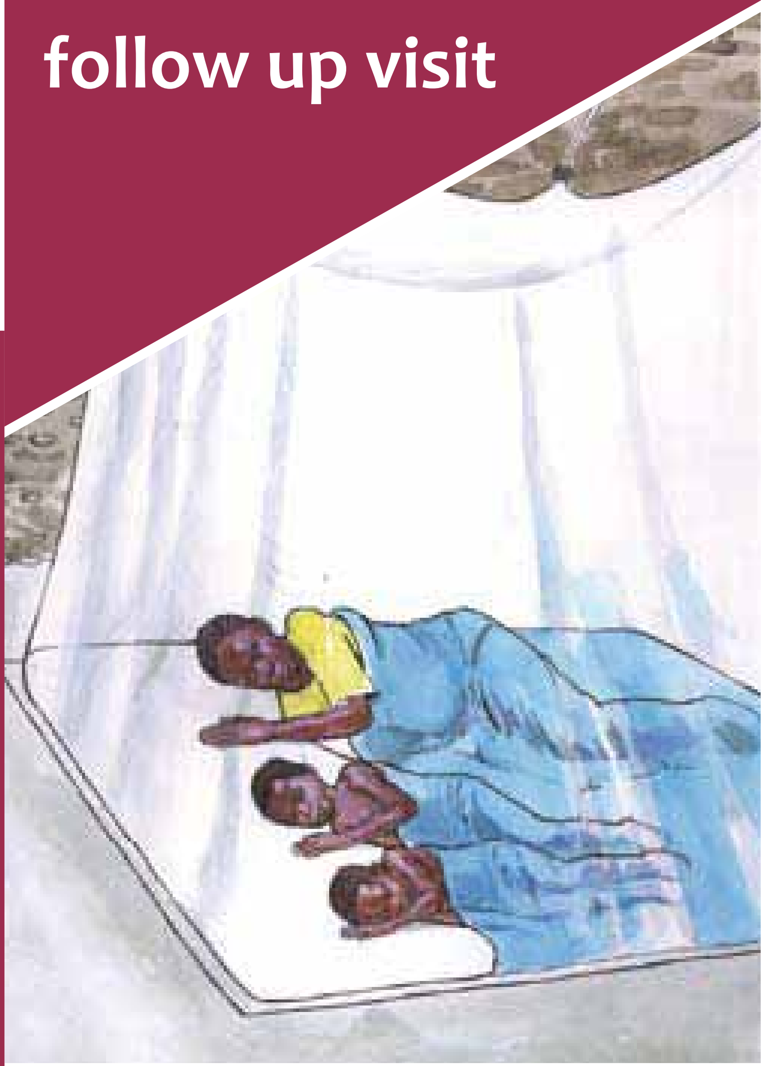

MEMBER'S NAME:

....

THE REPUBLIC OF UGANDA MINISTRY OF HEALTH

NetWorks

CDC USAID FROM THE AMERICAN PEOPLE

President's Malaria Initiative

BEFORE YOU SET OUT TO VISIT HOUSEHOLDS:

- Wear your VHT T-shirt, badge or any identifiable attire used by campaign team
- Ensure you are carrying enough 'hang-up follow-up' forms with you

How to introduce yourself

- Introduce yourself to each head of household (HH) or adult in the family and explain the purpose of your visit:

  'My name is ……. I'm a VHT member in the village ……. you were entitled to a free net ……. days/months ago and I'm here to follow up on that'

- Explain what data you are collecting and why
- Before asking to see the nets in the house, explain:

  'You know malaria is a deadly disease and sleeping under a net protects users. Since the last distribution, I want to see how many nets you have in your household now, if you have been able to hang and use them correctly and if there is any way I can help'

IF YOU ARE ALLOWED INSIDE THE HOUSE:

1. Record total number of nets in the house
2. Record actual number of nets in use (they may be hanging, folded up or knotted above the sleeping place or nearby)
3. If nets are already in use, congratulate them for using correctly!
4. If you see some are not in use, ask if you can help them to hang the nets and demonstrate the correct way to use. If they agree, help them put it up
5. Record any reasons for using nets (refer to section '8 reasons why everyone, every day, every night should use LLINs') for not using nets
6. Before leaving the house, ensure you recorded the total number of nets in the house:

   (a) You have seen hanging
   (b) Condition
   (c) Number you helped to hang

7. Finish filling the form
8. Encourage them to talk to neighbours about why it is important to use nets and benefits they have felt/seen
9. Remind them on how to best care for their LLINs (refer to section 'how to care for LLINs')
10. Thank them before you leave the house

IF YOU ARE NOT ALLOWED INSIDE THE HOUSE:

1. Record in the column on follow up form and ask head of the household "how many nets do you own and how many are you using now?"
2. If they tell you that some nets are not in use, go through the '8 reasons why everyone, every day, every night should use LLINs'
3. Record any reasons for not using nets
4. Finish filling the form
5. Ask if they have any more questions on LLINs
6. Encourage them to talk to neighbours about why it is important to use nets and benefits they have felt/seen
7. Remind them on how to best care for their LLINs (refer to section 'how to care for LLINs')
8. Thank them for their time and inform that a study team might follow up with a visit soon

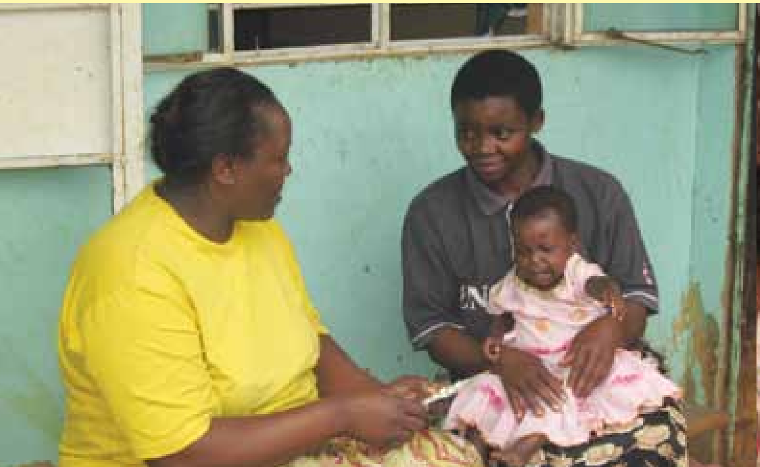

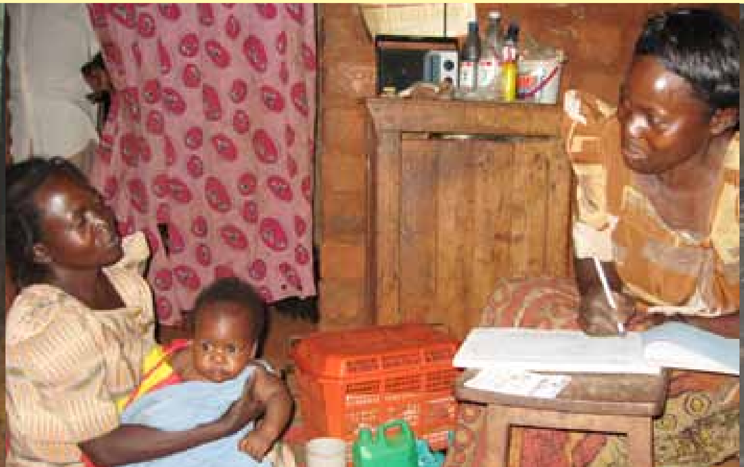

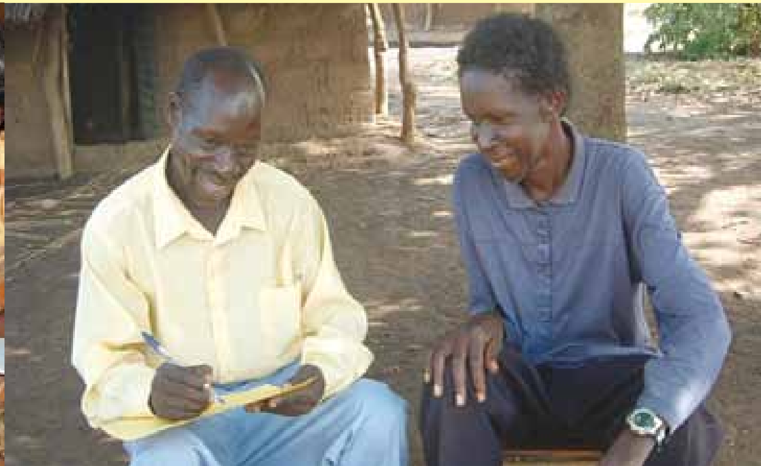

Supplement: S2 File — (PDF) [file pone.0119078.s002.pdf]
