# Supplementary material for: The Effect of Single or Repeated Home Visits on the Hanging and Use of Insecticide-Treated Mosquito Nets following a Mass Distribution Campaign - A Cluster Randomized, Controlled Trial
Source: PLoS One. 2015 Mar 16;10(3):e0119078. doi: 10.1371/journal.pone.0119078 (PMC4361725; doi:10.1371/journal.pone.0119078)
Supplement: S3 File — (DOCX) [file pone.0119078.s003.docx]

**Additional file S2: Composition of final sample by study arm and intervention cluster**

|  | **Survey 1**  **December 2010** | | **Survey 2**  **March 2011** | | **Survey 3**  **July 2011** | |
| --- | --- | --- | --- | --- | --- | --- |
|  | Households | % | Households | % | Households | % |
| **Arm 1: one visit** |  |  |  |  |  |  |
| Cluster 1 | 224 | 33.8% | 205 | 32.6% | 211 | 33.9% |
| Cluster 2 | 87 | 13.1% | 86 | 13.7% | 77 | 12.4% |
| Cluster 3 | 85 | 12.8% | 76 | 12.1% | 87 | 14.0% |
| Cluster 4 | 112 | 16.9% | 112 | 17.8% | 103 | 16.5% |
| Cluster 5 | 154 | 23.3% | 149 | 23.7% | 145 | 23.3% |
| **TOTAL** | **662** | **100%** | **628** | **100%** | **623** | **100%** |
| **Arm 2: two visits** |  |  |  |  |  |  |
| Cluster 1 | 184 | 27.5% | 180 | 28.7% | 171 | 28.9% |
| Cluster 2 | 132 | 19.8% | 123 | 19.6% | 115 | 19.5% |
| Cluster 3 | 95 | 14.2% | 91 | 14.5% | 87 | 14.7% |
| Cluster 4 | 67 | 10.0% | 68 | 10.8% | 67 | 11.3% |
| Cluster 5 | 190 | 28.4% | 166 | 26.4% | 151 | 25.5% |
| **TOTAL** | **668** | **100%** | **628** | **100%** | **591** | **100%** |
| **Arm 3: control** |  |  |  |  |  |  |
| Cluster 1 | 155 | 23.6% | 150 | 23.2% | 129 | 21.6% |
| Cluster 2 | 180 | 27.4% | 173 | 26.7% | 165 | 27.6% |
| Cluster 3 | 125 | 19.1% | 132 | 20.4% | 120 | 20.1% |
| Cluster 4 | 110 | 16.8% | 110 | 17.0% | 106 | 17.7% |
| Cluster 5 | 86 | 13.1% | 82 | 12.7% | 77 | 12.9% |
| **TOTAL** | **656** | **100%** | **647** | **100%** | **597** | **100%** |
