# Supplementary material for: The Effect of Single or Repeated Home Visits on the Hanging and Use of Insecticide-Treated Mosquito Nets following a Mass Distribution Campaign - A Cluster Randomized, Controlled Trial
Source: PLoS One. 2015 Mar 16;10(3):e0119078. doi: 10.1371/journal.pone.0119078 (PMC4361725; doi:10.1371/journal.pone.0119078)
Supplement: S4 File — (DOCX) [file pone.0119078.s004.docx]

**Additional file S4: Determinant of ITN use from multi-variable analysis.** Logistic regression of ITN use last night based on *de-facto* population of all three surveys; N=28,000

| Variable | Odds Ratio | 95% CI | p-value category | p-value variable |
| --- | --- | --- | --- | --- |
| Category |  |  |  |  |
| Intention to use net every or most nights | 3.22 | 2.75, 3.78 | - - | <0.0001 |
|  |  |  |  |  |
| At last 1 ITN for 2 people in household | 2.76 | 2.45, 3.12 | - - | <0.0001 |
|  |  |  |  |  |
| Confidence in preventive action (definitely could) | 1.23 | 1.10, 1.36 | - - | <0.0001 |
|  |  |  |  |  |
| Perception of threat of malaria (definitely agree) | 0.81 | 0.71, 0.92 | - - | <0.0001 |
|  |  |  |  |  |
| Discussed net use in family | 1.25 | 1.13, 1.38 | - - | 0.002 |
|  |  |  |  |  |
| Gender male vs. female | 1.17 | 1.11, 1.22 | - - | <0.001 |
|  |  |  |  |  |
| Age group in years 0-4 | 1.00 | - - | - - | <0.0001 |
| 5-9 | 0.52 | 0.48, 0.57 | <0.0001 |  |
| 10-14 | 0.27 | 0.24, 0.31 | <0.0001 |  |
| 15-19 | 0.26 | 0.23, 0.30 | <0.0001 |  |
| 20-29 | 0.66 | 0.58, 0.75 | <0.0001 |  |
| 30-39 | 0.86 | 0.75, 0.98 | 0.03 |  |
| 40-49 | 0.62 | 0.51, 0.75 | <0.0001 |  |
| 50-59 | 0.56 | 0.47, 0.68 | <0.0001 |  |
| 60+ | 0.35 | 0.28, 0.43 | <0.0001 |  |
|  |  |  |  |  |
| Wealth quintiles lowest | 1.00 | - - | - - | <0.0001 |
| Second | 0.86 | 0.72, 1.02 | 0.09 |  |
| Third | 0.75 | 0.63, 0.88 | 0.001 |  |
| Forth | 0.73 | 0.61, 0.88 | 0.001 |  |
| highest | 0.61 | 0.50, 0.75 | <0.0001 |  |
|  |  |  |  |  |
| Education of head of household no school | 1.00 | - - | - - | 0.11 |
| Primary school | 1.12 | 0.98, 1.29 | 0.09 |  |
| Secondary or better | 1.15 | 1.00, 1.31 | 0.04 |  |
|  |  |  |  |  |
| Household size (de jure) 1-3 | 1.00 | - - | - - | 0.10 |
| 4-6 | 0.81 | 0.67, 0.99 | 0.04 |  |
| 7-10 | 0.90 | 0.73, 1.09 | 0.26 |  |
| 11+ | 0.88 | 0.69, 1.12 | 0.29 |  |
|  |  |  |  |  |
| Surveys Survey 1 | 1.00 | - - | - - | 0.04 |
| Survey 2 | 1.24 | 1.02, 1.51 | 0.03 |  |
| Survey 3 | 1.38 | 1.02, 1.85 | 0.04 |  |
|  |  |  |  |  |
| Study arm One visit (arm 1) | 1.00 | - - | - - | 0.06 |
| Two visits (arm 2) | 1.07 | 0.85, 1.35 | 0.57 |  |
| Control (arm 3) | 0.84 | 0.58, 1.20 | 0.32 |  |
|  |  |  |  |  |
